# Cardiotoxicity from intensive chemotherapy combined with radiotherapy in breast cancer.

**DOI:** 10.1038/bjc.1997.489

**Published:** 1997

**Authors:** H. de Graaf, W. V. Dolsma, P. H. Willemse, W. T. van der Graaf, D. T. Sleijfer, E. G. de Vries, N. H. Mulder

**Affiliations:** Department of Internal Medicine, University Hospital Groningen, The Netherlands.

## Abstract

Cardiac function was evaluated in 86 breast cancer patients after standard chemotherapy, followed by ablative chemotherapy and chest irradiation. One patient died of subacute heart failure 3 months after ablative chemotherapy. At a minimum of 1 year's follow-up (range 1-11 years) left vertricular ejection fraction (LVEF) was marginally abnormal in 4 of 27 disease-free survivors. One exceptional patient who received two transplantations is alive, with serious heart failure occurring after the second ablative chemotherapy. Including this patient, the percentage of patients free of clinical and subclinical cardiac dysfunction at 7 years is 78% (95% CI 61-95%). After ablative chemotherapy, cardiotoxicity was rarely life-threatening. The impact of subclinical cardiotoxicity in the long term is not clear and needs continued evaluation.


British Journal of Cancer (1997) 76(7), 943–945
© 1997 Cancer Research Campaign

### Cardiotoxicity from intensive chemotherapy combined with radiotherapy in breast cancer

H de Graaf[1], WV Dolsma[2], PHB Willemse[1], WTA van der Graaf[1], DTh Sleijfer[1], EGE de Vries[1] and NH Mulder[1]

[1]Division of Medical Oncology, Department of Internal Medicine; [2] Radiotherapy, University Hospital Groningen, The Netherlands

**Summary** Cardiac function was evaluated in 86 breast cancer patients after standard chemotherapy, followed by ablative chemotherapy and chest irradiation. One patient died of subacute heart failure 3 months after ablative chemotherapy. At a minimum of 1 year's follow-up (range 1–11 years) left vertricular ejection fraction (LVEF) was marginally abnormal in 4 of 27 disease-free survivors. One exceptional patient who received two transplantations is alive, with serious heart failure occurring after the second ablative chemotherapy. Including this patient, the percentage of patients free of clinical and subclinical cardiac dysfunction at 7 years is 78% (95% CI 61–95%). After ablative chemotherapy, cardiotoxicity was rarely life-threatening. The impact of subclinical cardiotoxicity in the long term is not clear and needs continued evaluation.

**Keywords:** ablative chemotherapy; breast cancer; cardiotoxicity; radiotherapy; autologous transplantation

Intensive chemotherapy, which we have defined by induction chemotherapy followed by ablative chemotherapy with bone marrow or stem cell rescue, combined with radiotherapy is, at present, a usual treatment for patients with locally advanced or disseminated breast cancer (Triozzi et al, 1995). Many chemotherapeutic drugs and radiotherapy have toxic effects on the heart. The anthracyclines are notorious for their cumulative cardiotoxicity (Von Hoff et al, 1979; Cornbleet et al, 1984; Nielsen et al, 1990). Late cardiotoxicity can occur more than 1 year after drug exposure (Rhoden et al, 1993; Steinherz et al, 1995). Subacute cardiotoxicity is considered to be reversible, whereas late effects are considered to be lasting and progressive with time (Cohen et al, 1982; Saini et al, 1987; Shapiro and Henderson, 1994). Radiation-induced heart disease (RIHD) includes late congestive heart failure (CHF) or coronary artery disease (Tötterman et al, 1983; Cuzick et al, 1994; Gyenes et al, 1994; Stewart et al, 1995). The risk of RIHD is related to the total radiation dose and the irradiated volume (Steinherz and Yahalom, 1993; Gyenes et al, 1994). There are reports that left-sided chest irradiation causes more cardiotoxicity than right-sided irradiation (Rutqvist et al, 1992).

Data about late cardiotoxic effects of intensive chemotherapy, combined with radiotherapy in the treatment of breast cancer, are limited. Therefore, we have evaluated the cardiac function in breast cancer patients several years after standard-dose doxorubicin- or epirubicin-based induction therapy, followed by cyclophosphamide- or mitoxantrone-based ablative therapy and irradiation. Cardiac function was evaluated by measuring left ventricular ejection fraction (LVEF) by means of a radionuclide angiography, because it is still considered to be the standard non-invasive method for detecting cardiotoxicity (McKillop et al, 1983; Druck et al, 1984; John et al, 1988).

*Received 18 June 1996*
*Revised 26 March 1997*
*Accepted 2 April 1997*

*Correspondence to:* H de Graaf, Division of Medical Oncology, Department of Internal Medicine, University Hospital, PO Box 30.001, 9700 RB Groningen, The Netherlands

### PATIENTS AND METHODS

Eighty-six premenopausal patients with locally advanced or disseminated breast cancer were treated with intensive (induction and ablative) chemotherapy supported by bone marrow or stem cell rescue and radiotherapy between 1984 and 1994. Before treatment, none of the patients had manifest signs or symptoms of cardiac disease. Fifty-two patients (60%) were alive at the start of a cardiac evaluation in September 1994. Cardiac function was assessed in 27 of these 52 patients, who were more than 1 year in follow-up after completing intensive chemotherapy. The median follow-up of the study group was 2 years (range 1–11 years); their median age was 42 years (range 28–54 years).

The chemotherapy consisted of doxorubicin- or epirubicin-based standard induction chemotherapy, followed by cyclophosphamide- or mitoxantrone-based ablative chemotherapy. Twenty-four patients received a cumulative dose of doxorubicin of 300 mg $m^{-2}$ intravenously (De Graaf et al, 1994). Three patients received a cumulative dose of epirubicin of 480 mg $m^{-2}$. Six patients received a cyclophosphamide-based ablative regimen with a cumulative dose of 7 g $m^{-2}$ cyclophosphamide given intravenously over three consecutive days in combination with 1.5 g $m^{-2}$ etoposide (CE). Twenty patients received a mitoxantrone-based ablative regimen: in 14 patients, 50 mg $m^{-2}$ mitoxantrone was combined with 800 mg $m^{-2}$ thiotepa (MT); in three patients, 60 mg $m^{-2}$ mitoxantrone was combined with 180 mg $m^{-2}$ melphalan (MM); and in three patients, 800 mg $m^{-2}$ thiotepa (TMM) was added. One patient had both CE and the TMM regimen.

Seventeen of 20 patients with locally advanced disease received 50–66 Gy locoregional radiotherapy a median of 11 weeks (range 8–19) after the ablative chemotherapy. In four of these 17 patients, this was part of a breast-conserving treatment. Of seven patients with disseminated disease, six received radiotherapy – four as primary treatment before chemotherapy and two patients after the

**Table 1** Effect of ablative chemotherapy (after pretreatment with anthracycline-based induction chemotherapy) on LVEF of 26 breast cancer patients without clinically manifest cardiotoxicity

| Ablative chemotherapy | Number of patients | Abnormal LVEF (< 55%) n (%) |
|---|---|---|
| CE | 6 | 1 (17) |
| MT | 14 | 1 (7) |
| MM | 3 | 1 (33) |
| TMM | 3 | 1 (33) |
| Total | 26 | 4 |

CE, cyclophosphamide 7 g $m^{-2}$ + etoposide 1.5 g $m^{-2}$; MT, mitoxantrone 50 mg $m^{-2}$ + thiotepa 800 mg $m^{-2}$; MM, mitoxantrone 60 mg $m^{-2}$ + melphalan 180 mg $m^{-2}$; TMM, mitoxantrone 60 mg $m^{-2}$ + melphalan 180 mg $m^{-2}$ + thiotepa 800 mg $m^{-2}$.

**Figure 1** Percentage of patients free of clinical and subclinical cardiac dysfunction out of 86 patients treated with high-dose chemotherapy and radiotherapy. The median observation (arrow) is 24 months. The percentage of patients free of cardiac dysfunction at 84 months is 78% (95% CI 61–95%)

ablative chemotherapy. In all cases irradiation was given with 4–6 MeV photons from a linear accelerator in fractions of 2 Gy to the breast or chest wall, the supraclavicular, axillary and internal mammary lymph nodes. The internal mammary lymph nodes were irradiated by a direct anterior field after amputation and by a wedged tangential field for breast-conserving treatment.

Cardiac evaluation consisted of clinical functional classification according to the New York Heart Association (NYHA), standard 12-lead electrocardiogram (ECG) and left ventricular ejection fraction (LVEF) by ECG-gated radionuclide angiography. For the calculation of the LVEF, 400-MBq $^{99m}$Tc-labelled autologous red blood cells were injected and acquisition was performed in 6 min with a large-field-of-view gamma-camera with a low-energy all-purpose parallel-hole collimator. A LVEF of < 55% is the lower cut-off value in our hospital.

Statistical analysis was performed by the chi-square (Mantel–Haenszel) and Fisher exact test, and survival curves were plotted using the Kaplan–Meier method.

### RESULTS

Of the 86 patients who were treated with intensive chemotherapy and irradiation for breast cancer, 34 had died before the start of the cardiac evaluation in September 1994, leaving 52 patients at risk.

**Figure 2** Overall survival in 86 patients treated with high-dose chemotherapy and radiotherapy. The median observation (arrow) is 24 months. The overall survival at 84 months is 45% (95% CI 33–58%)

In one of these patients, the cause of death was subacute cardiotoxicity 3 months after the ablative chemotherapy. This concerned a 42-year-old patient treated with cumulative 300 mg $m^{-2}$ doxorubicin and 50 mg $m^{-2}$ mitoxantrone without radiotherapy, who was admitted to the hospital because of rapidly progressive CHF. The echocardiogram showed a markedly decreased contractility, without pericardial effusion. She had no pre-existing heart disease. Post-mortem examination was denied.

One of the 27 patients received a double transplant with a 4-year interval. She developed heart failure 2 weeks after her second transplant. The LVEF dropped from 58% to 13%. With diuretics, digoxin, dobutamine and an ACE inhibitor, the clinical situation is stable and acceptable at this moment (2 years later). Functionally, she is categorized as stage III NYHA.

The other 26 patients had no clinical signs or symptoms of cardiotoxicity (NYHA classification I). The QTc interval was normal in all 26 patients. The LVEF was slightly abnormal in four patients treated with TMM, MT, MM and CE ablative chemotherapy (50% in one and 52% in three patients) after 1, 3, 6 and 7 years follow-up respectively (Table 1). The abnormal LVEF occurred in two patients after left- and, in two, after right-sided irradiation. Because of this small number, no conclusions on the influence of left- vs right-sided irradiation or the effect of combination radio-chemotherapy is possible. We estimated the percentage of patients free of cardiac dysfunction at 5 and 7 years, adding the two clinical and four subclinical events. As shown in Figure 1, at 5 years the estimated percentage of patients free of cardiac dysfunction is 94% (95% CI 88–100%) and at 7 years 78% (95% CI 61–95%) (Figure 1). The overall survival is shown in Figure 2.

### DISCUSSION

In the treatment of breast cancer, in most cases, a combination of chemotherapy and radiotherapy is used. In patients treated with 'standard' adjuvant chemotherapy with a cumulative dose of 300 mg $m^{-2}$ doxorubicin and left-sided irradiation, 2.6% CHF was found after a median follow-up of 6.5 years, compared with 0.3% CHF for right-sided or no irradiation (Valagussa et al, 1994).

Little is known about the acute and late cardiotoxic effects of intensive chemotherapy, which represents a cumulative effect of standard and ablative chemotherapy, and of its combination with

© Cancer Research Campaign 1997

chest irradiation. All ablative regimens were based on cyclophosphamide (CE) or mitoxantrone (MT, MM or TMM). Cyclophosphamide can induce acute cardiotoxicity, especially in children, but it is unclear whether it can cause late cardiotoxicity (Gottdiener et al, 1981; Steinherz and Yahalom, 1993). In 18 adults with haematological diseases (mean age 35 years) treated with high-dose cyclophosphamide (5.5–8 g $m^{-2}$), only one patient (5.5%) developed a mild CHF with decrease of LVEF to 40% (Braverman et al, 1991). For the CE combination, we have reported the results of a phase I study using 7 g $m^{-2}$ cyclophosphamide and 2–2.5 g $m^{-2}$ etoposide without radiotherapy. No acute cardiotoxicity was observed in six patients (Mulder et al, 1984). Mitoxantrone given after anthracyclines in an ablative regimen in a cumulative dose of 100 mg $m^{-2}$ may lead to 3% CHF (Shenkenberg and Von Hoff, 1986). In the study of Bowers et al (1993), 44 breast cancer patients were treated with escalating doses of mitoxantrone in combination with 900 mg $m^{-2}$ thiotepa after pretreatment with 160–492 mg $m^{-2}$ doxorubicin. No acute cardiotoxicity was observed using 50 mg $m^{-2}$ mitoxantrone in 13 patients whereas, after 60 mg $m^{-2}$ mitoxantrone, CHF occurred in 4 out of 31 patients (13%). One patient developed acute pericarditis and four patients had a $\geq$ 10% decline in LVEF, amounting to an overall incidence of acute cardiotoxicity of 29% (Bowers et al, 1993). In the present study, we found that life-threatening cardiotoxicity is a rare complication and late subclinical cardiotoxicity was found in 4 of 27 evaluated patients at a median 2 years' follow-up, which occurred in all ablative regimens and was not restricted to a particular schedule. The estimated percentage of patients free of cardiac dysfunction, including the two clinical and four subclinical events, at 5 years is 94% (95% CI 88–100%) and at 7 years 78% (95% CI 61–95%). These results are hampered by a limited follow-up and a small number of events, although an increasing incidence of cardiac dysfunction at longer follow-up is suggested. Important is the recognition that the patients treated with intensive chemotherapy are, in general, young adults without a pre-existent cardiac history. Because of the curative intent of the intensive treatment, the evaluation of cardiotoxicity is of importance for the future quality of life of these patients. The risk of long-term cardiotoxicity is especially important in the adjuvant setting and is much more of a theoretical concern in the therapy of metastatic disease as, if these patients should live long enough to experience such late effects, this would be regarded as a victory for the treatment.

In conclusion, thus far, the low incidence of clinical and subclinical cardiotoxicity in adult breast cancer patients does not hamper the use of intensive chemotherapy combined with radiotherapy in patients who would otherwise have a poor chance of survival.

### REFERENCES

Bowers C, Adkins D, Dunphy F, Harrison B, Lemaistre CF and Spitzer G (1993) Dose escalation of mitoxantrone given with thiotepa and autologous bone marrow transplantation for metastatic breast cancer. *Bone Marrow Transplant* **12**: 525–530

Braverman AC, Antin JH, Plappert MT, Cook EF and Lee RT (1991) Cyclophosphamide cardiotoxicity in bone marrow transplantation: a prospective evaluation of new dosing regimens. *J Clin Oncol* **9**: 1215–1223

Cohen M, Kronzon I and Lebowitz A (1982) Reversible doxorubicin-induced congestive heart failure. *Arch Int Med* **142**: 1570–1571

Cornbleet MA, Stuart-Harris RC, Smith IE, Coleman RE, Rubens RD, McDonald M, Mouridsen HT, Rainer H, van Oosterom AT and Smyth JF (1984) Mitoxantrone for the treatment of advanced breast cancer: single-agent therapy in previously untreated patients. *Eur J Cancer Clin Oncol* **20**: 1141–1146

Cuzick J, Stewart H, Rutqvist L, Houghton J, Edwards R, Redmond C, Peto R, Baum M, Fisher B, Host H, Lythgoe J, Ribeiro G and Scheurlen H (1994) Cause-specific mortality in long-term survivors of breast cancer who participated in trials of radiotherapy. *J Clin Oncol* **12**: 447–453

De Graaf H, Willemse PHB, Sleijfer DTh, De Vries EGE, Van der Graaf WTA, Beukema J and Mulder NH (1994) Effective conditioning regimen for premenopausal patients with advanced breast cancer. *Anticancer Res* **14**: 2799–2804

Druck MN, Gulenchyn KY, Evans WK, Gotlieb A, Srigley JR, Bar-Shlomo BZ, Feiglin DH, McEwan P, Silver MD, Millband L, Winter K, Hilton JD, Jablonsky G, Morch JE and McLaughlin P (1984) Radionuclide angiography and endomyocardial biopsy in the assessment of doxorubicin cardiotoxicity. *Cancer* **53**: 1667–1674

Gottdiener JS, Appelbaum FR, Ferrans VJ, Deisseroth A and Ziegler J (1981) Cardiotoxicity associated with high-dose cyclophosphamide therapy. *Arch Int Med* **141**: 758–763

Gyenes G, Fornander T, Carlens P and Rutqvist LE (1994) Morbidity of ischemic heart disease in early breast cancer 15–20 years after adjuvant radiotherapy. *Int J Rad Oncol Biol Phys* **28**: 1235–1241

John V, Muller RD, Reiners C and Lohr E (1988) Radiological diagnosis of late effects on thoracic organs after chemotherapy and/or radiation therapy as well as after radionuclide therapy. *Strahlenther Onkol* **164**: 619–628

McKillop JH, Bristow MR, Goris ML, Billingham ME and Bockemuehl K (1983) Sensitivity and specificity of radionuclide ejection fractions in doxorubicin cardiotoxicity. *Am Heart J* **106**: 1048–1056

Mulder NH, Meinesz AF, Sleijfer DTh, Postmus PE, De Vries EGE, Van der Geest S, Orie JLM and Vriesendorp R (1984) Feasibility of high dose VP 16–213 as single agent or in combination with cyclophosphamide and autologous bone marrow transplantation (ABMT). *Neth J Med* **27**: 389–392

Nielsen D, Jensen JB, Dombernowsky P, Munck O, Fogh J, Brynjolf I, Havsteen H and Hanssen M (1990) Epirubicin cardiotoxicity: a study of 135 patients with advanced breast cancer. *J Clin Oncol* **8**: 1806–1810

Rhoden W, Hasleton P and Brooks N (1993) Anthracyclines and the heart. *Br Heart J* **70**: 499–502

Rutqvist LE, Lax I, Fornander T and Johansson H (1992) Cardiovascular mortality in a randomized trial of adjuvant radiation therapy versus surgery alone in primary breast cancer. *Int J Rad Oncol Biol Phys* **22**: 887–896

Saini J, Rich MW and Lyss AP (1987) Reversibility of severe left ventricular dysfunction due to doxorubicin cardiotoxicity. *Ann Int Med* **106**: 814–816

Shapiro CL and Henderson IC (1994) Late cardiac effects of adjuvant therapy: too soon to tell? *Ann Oncol* **5**: 196–198

Shenkenberg TD and Von Hoff DD (1986) Mitoxantrone: a new anticancer drug with significant clinical activity. *Ann Int Med* **105**: 67–81

Steinherz LJ and Yahalom J (1993) Cardiac complications of cancer therapy. In *Cancer Principles and Practice of Oncology*, De Vita VT, Hellman S and Rosenberg SA. (eds), pp. 2370–2385. Lippincott: Philadelphia

Steinherz LJ, Steinherz PG and Tan C (1995) Cardiac failure and dysrhythmias 6–19 years after anthracycline therapy: a series of 15 patients. *Med Pediatr Oncol* **24**: 352–361

Stewart JR, Fajardo LF, Gillette SM and Constine LS (1995) Radiation injury to the heart. *Int J Rad Oncol Biol Phys* **31**: 1205–1211

Tötterman KJ, Pesonen E and Siltanen P (1983) Radiation-related chronic heart disease. *Chest* **83**: 875–878

Triozzi PL, Rhoades C and Thornton DE (1995) High-dose chemotherapy for breast cancer. *Cancer Treat Rev* **21**: 185–198

Valagussa P, Zambetti M, Biasi S, Moliterni A, Zucali R and Bonadonna G (1994) Cardiac effects following adjuvant chemotherapy and breast irradiation in operable breast cancer. *Ann Oncol* **5**: 209–216

Von Hoff DD, Layard MW, Basa P, Davis Jr HL, Von Hoff AL, Rozencweig M and Muggia FM (1979) Risk factors for doxorubicin-induced congestive heart failure. *Ann Int Med* **91**: 710–719

© Cancer Research Campaign 1997


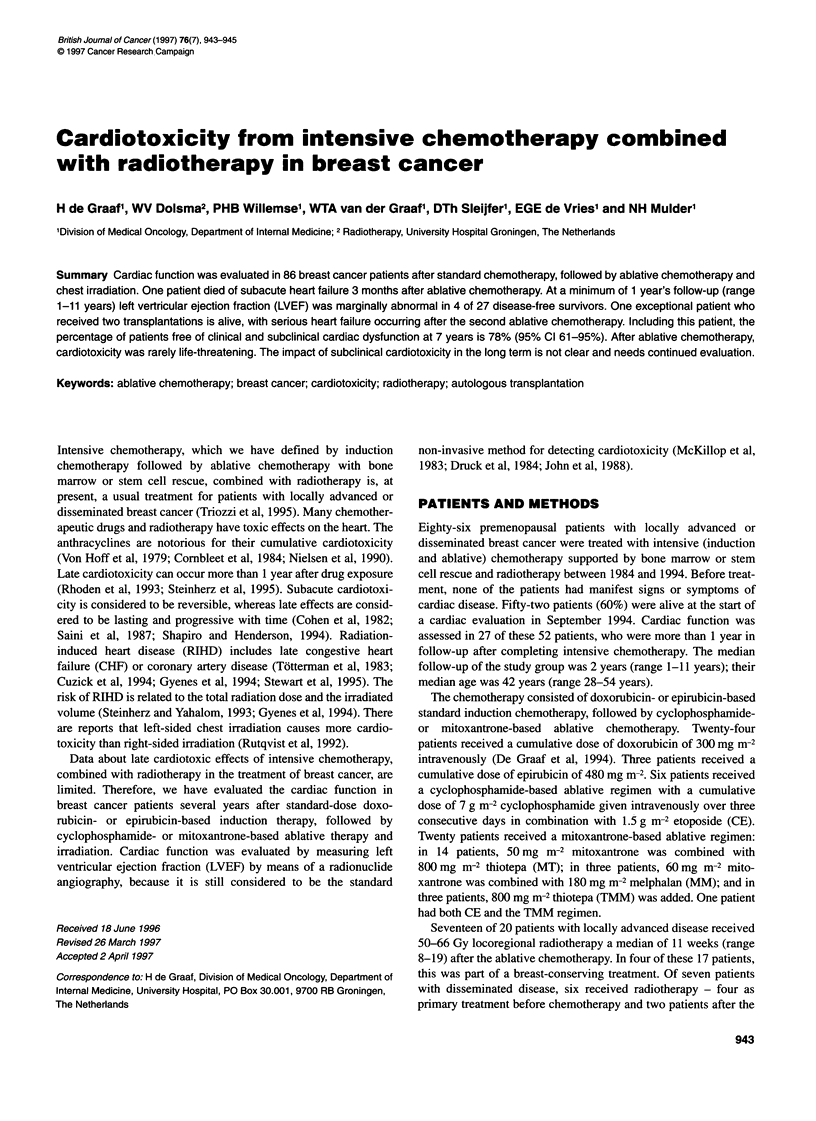

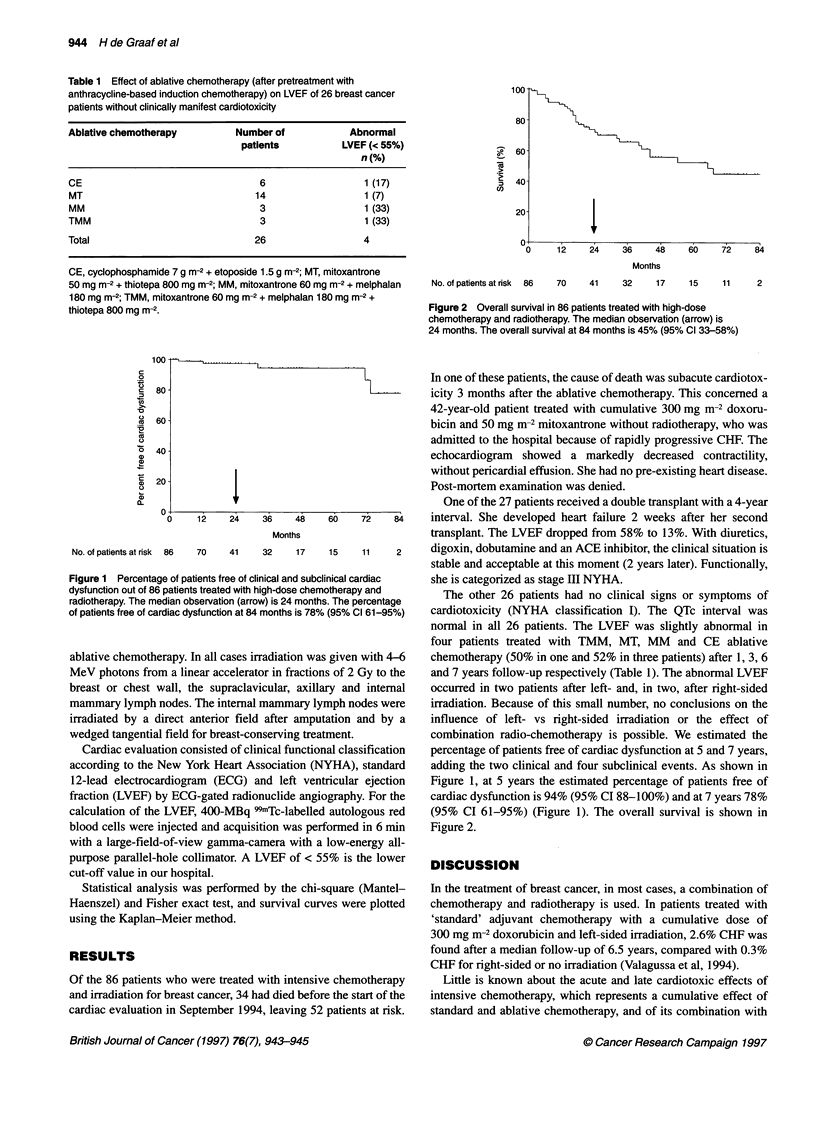

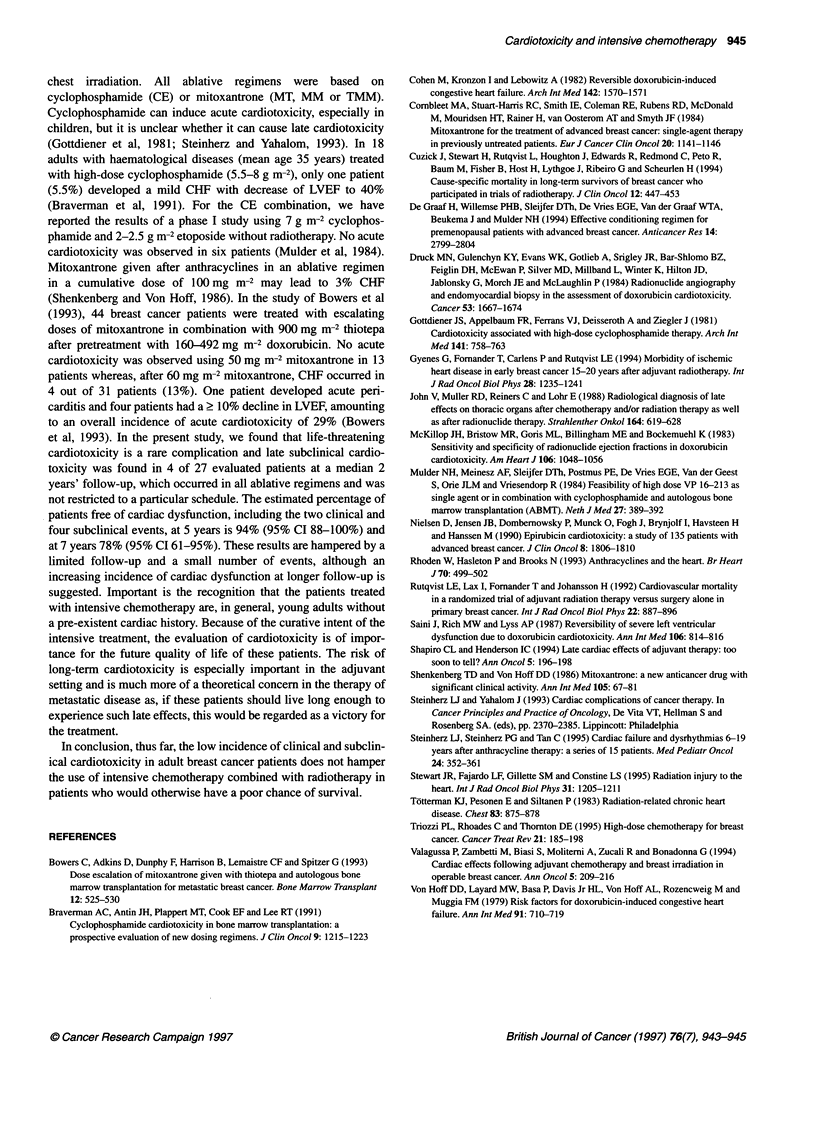


## References

[OCR_00328] Bowers C., Adkins D., Dunphy F., Harrison B., LeMaistre C. F., Spitzer G. (1993). Dose escalation of mitoxantrone given with thiotepa and autologous bone marrow transplantation for metastatic breast cancer.. Bone Marrow Transplant.

[OCR_00335] Braverman A. C., Antin J. H., Plappert M. T., Cook E. F., Lee R. T. (1991). Cyclophosphamide cardiotoxicity in bone marrow transplantation: a prospective evaluation of new dosing regimens.. J Clin Oncol.

[OCR_00341] Cohen M., Kronzon I., Lebowitz A. (1982). Reversible doxorubicin-induced congestive heart failure.. Arch Intern Med.

[OCR_00345] Cornbleet M. A., Stuart-Harris R. C., Smith I. E., Coleman R. E., Rubens R. D., McDonald M., Mouridsen H. T., Rainer H., van Oosterom A. T., Smyth J. F. (1984). Mitoxantrone for the treatment of advanced breast cancer: single-agent therapy in previously untreated patients.. Eur J Cancer Clin Oncol.

[OCR_00351] Cuzick J., Stewart H., Rutqvist L., Houghton J., Edwards R., Redmond C., Peto R., Baum M., Fisher B., Host H. (1994). Cause-specific mortality in long-term survivors of breast cancer who participated in trials of radiotherapy.. J Clin Oncol.

[OCR_00364] Druck M. N., Gulenchyn K. Y., Evans W. K., Gotlieb A., Srigley J. R., Bar-Shlomo B. Z., Feiglin D. H., McEwan P., Silver M. D., Millband L. (1984). Radionuclide angiography and endomyocardial biopsy in the assessment of doxorubicin cardiotoxicity.. Cancer.

[OCR_00372] Gottdiener J. S., Appelbaum F. R., Ferrans V. J., Deisseroth A., Ziegler J. (1981). Cardiotoxicity associated with high-dose cyclophosphamide therapy.. Arch Intern Med.

[OCR_00377] Gyenes G., Fornander T., Carlens P., Rutqvist L. E. (1994). Morbidity of ischemic heart disease in early breast cancer 15-20 years after adjuvant radiotherapy.. Int J Radiat Oncol Biol Phys.

[OCR_00382] John V., Müller R. D., Reiners C., Löhr E. (1988). Radiologisch-diagnostische Aspekte zu Späteffekten an den Thoraxorganen nach Chemo- und/oder Strahlentherapie sowie nach Behandlung mit Radionukliden.. Strahlenther Onkol.

[OCR_00387] McKillop J. H., Bristow M. R., Goris M. L., Billingham M. E., Bockemuehl K. (1983). Sensitivity and specificity of radionuclide ejection fractions in doxorubicin cardiotoxicity.. Am Heart J.

[OCR_00392] Mulder N. H., Meinesz A. F., Sleijfer D. T., Postmus P. E., De Vries E. G., Van der Geest S., Orie J. L., Vriesendorp R. (1984). Feasibility of high dose VP 16-213 as single agent or in combination with cyclophosphamide and autologous bone marrow transplantation (ABMT).. Neth J Med.

[OCR_00398] Nielsen D., Jensen J. B., Dombernowsky P., Munck O., Fogh J., Brynjolf I., Havsteen H., Hansen M. (1990). Epirubicin cardiotoxicity: a study of 135 patients with advanced breast cancer.. J Clin Oncol.

[OCR_00403] Rhoden W., Hasleton P., Brooks N. (1993). Anthracyclines and the heart.. Br Heart J.

[OCR_00407] Rutqvist L. E., Lax I., Fornander T., Johansson H. (1992). Cardiovascular mortality in a randomized trial of adjuvant radiation therapy versus surgery alone in primary breast cancer.. Int J Radiat Oncol Biol Phys.

[OCR_00412] Saini J., Rich M. W., Lyss A. P. (1987). Reversibility of severe left ventricular dysfunction due to doxorubicin cardiotoxicity. Report of three cases.. Ann Intern Med.

[OCR_00416] Shapiro C. L., Henderson I. C. (1994). Late cardiac effects of adjuvant therapy: too soon to tell?. Ann Oncol.

[OCR_00420] Shenkenberg T. D., Von Hoff D. D. (1986). Mitoxantrone: a new anticancer drug with significant clinical activity.. Ann Intern Med.

[OCR_00429] Steinherz L. J., Steinherz P. G., Tan C. (1995). Cardiac failure and dysrhythmias 6-19 years after anthracycline therapy: a series of 15 patients.. Med Pediatr Oncol.

[OCR_00434] Stewart J. R., Fajardo L. F., Gillette S. M., Constine L. S. (1995). Radiation injury to the heart.. Int J Radiat Oncol Biol Phys.

[OCR_00442] Triozzi P. L., Rhoades C., Thornton D. E. (1995). High-dose chemotherapy for breast cancer.. Cancer Treat Rev.

[OCR_00438] Tötterman K. J., Pesonen E., Siltanen P. (1983). Radiation-related chronic heart disease.. Chest.

[OCR_00446] Valagussa P., Zambetti M., Biasi S., Moliterni A., Zucali R., Bonadonna G. (1994). Cardiac effects following adjuvant chemotherapy and breast irradiation in operable breast cancer.. Ann Oncol.

[OCR_00451] Von Hoff D. D., Layard M. W., Basa P., Davis H. L., Von Hoff A. L., Rozencweig M., Muggia F. M. (1979). Risk factors for doxorubicin-induced congestive heart failure.. Ann Intern Med.

[OCR_00359] de Graaf H., Willemse P. H., Sleijfer D. T., de Vries E. G., Van der Graaf W. T., Beukema J., Mulder N. H. (1994). Effective conditioning regimen for premenopausal patients with advanced breast cancer.. Anticancer Res.

